# Transitional experiences of mothers of children with special health needs in breastfeeding

**DOI:** 10.1590/0034-7167-2024-0272

**Published:** 2025-08-25

**Authors:** Ana Carla Silveira de Sá, Fernanda Garcia Bezerra Góes, Andressa Neto Souza, Laura Johanson da Silva, Aline Cerqueira Santos Santana da Silva, Juliana Rezende Montenegro Medeiros de Moraes, Liliane Faria da Silva, Fernando Rocha Porto

**Affiliations:** I Universidade Federal do Estado do Rio de Janeiro. Rio de Janeiro, Rio de Janeiro, Brazil.; II Universidade Federal Fluminense. Rio das Ostras, Rio de Janeiro, Brazil.; III Universidade Federal do Rio de Janeiro. Rio de Janeiro, Rio de Janeiro, Brazil.; IV Universidade Federal Fluminense. Rio de Janeiro, Rio de Janeiro, Brazil.

**Keywords:** Child, Disabled Children, Mothers, Breast Feeding, Chronic Disease, Niño, Niños con Discapacidad, Madres, Lactancia Materna, Enfermedad Crónica

## Abstract

**Objective:**

to describe the experiences of mothers of children with special health needs regarding breastfeeding in light of Meleis’s Transitions Theory.

**Methods:**

a qualitative study, using nine Sensitive Creative Method Knowledge Tree dynamics, with 20 mothers, in March 2023. Data were processed using the software Interface de R pour les Analyses Multidimensionnelles de Textes et de Questionnaires and subjected to thematic analysis.

**Results:**

mothers experienced multiple, complex, and simultaneous transitions, including aspects related to prematurity and intensive care hospitalization. Many faced emotional, physical, and social challenges while trying to breastfeed their infants in the face of adverse circumstances. One facilitator was the support of healthcare professionals at the milk bank.

**Final considerations:**

joint action between nurses and other healthcare professionals with mothers is relevant for quality transitional care in breastfeeding for children with special health needs.

## RESUMEN

**Objetivo:**

describir las experiencias de madres de niños con necesidades especiales de salud respecto a la lactancia materna a la luz de la Teoría de las Transiciones de Meleis.

**Métodos:**

estudio cualitativo, utilizando nueve dinámicas del Árbol del Conocimiento del Método Creativo Sensible, con 20 madres, en marzo de 2023. Los datos fueron procesados en el software Interface de R pour les Analyses Multidimensionnelles de Textes et de Questionnaires y sometidos a análisis temático.

**Resultados:**

las madres vivieron transiciones múltiples, complejas y simultáneas, abarcando aspectos relacionados con la prematuridad y la hospitalización en cuidados intensivos. Muchas enfrentaron desafíos emocionales, físicos y sociales al intentar amamantar a sus hijos ante circunstancias adversas. Un facilitador fue el apoyo de los profesionales de la salud en el banco de leche.

**Consideraciones finales:**

la acción conjunta entre enfermeras y otros profesionales de la salud con las madres es relevante para una atención transicional de calidad en la lactancia materna para niños con necesidades especiales de salud.

## INTRODUCTION

The global rate of exclusive breastfeeding (EBF) in the first four months of life is 35%, and in Brazil, the mean EBF is only 23 days^(1)^. The Brazilian Study of Infant Feeding and Nutrition revealed that, between February 2019 and March 2020, the prevalence of breastfeeding (BF) in the first hour of life was 62.4%, with a higher prevalence in the North region (73.5%), followed by the Midwest (64.0%) and Northeast (63.2%) regions, while the South (61.8%) and Southeast (58.5%) regions had the lowest prevalence. Furthermore, the prevalence of EBF in children under 6 months was 45.8%, also below international recommendations^(2)^.

BF of children with special healthcare needs (CSHCN) tends to be even shorter compared to healthy children due to different care demands, which contributes to early weaning. For instance, children with Down syndrome are breastfed less frequently than those without Down syndrome^(3)^. Furthermore, maintaining BF is more challenging in premature and at-risk newborns than in healthy full-term newborns, due to factors such as underlying pathology, clinical fragility, prolonged hospitalization, separation between mother and baby, and maternal stress^(4)^.

The birth of a child and BF are transitional events marked by several changes in the lives of those involved, with distinct implications and adaptations for the mother, baby and family. A mother’s transition to BF involves the transformation of her body, which begins during pregnancy. The body, previously exclusively hers, becomes a source of food for children. Therefore, this transi- tion involves moving from one stable state (place or condition) to another, requiring them to incorporate new knowledge, change their behaviors and change the definition of self (identity). The transition is healthy when there is mastery of new skills and behaviors necessary to manage the new life situation as well as the construction of a new identity^(5)^.

The complexity of all these transitions is amplified when mothers experience having a child with special health needs and breastfeed them. In this context, it is crucial to understand the transitions in terms of their nature, timing, severity and personal, family and social expectations^(5)^. The Transition Theory^(6)^ emerges as an essential theoretical basis for deepening the analysis of the transitional process experienced by mothers of CSHCN during BF. This understanding is essential to guide nurses’ transitional care, benefiting a healthy transition for this population. By offering support based on the real needs and interests of those involved, this professional acts to promote autonomy and well-being, contributing to the reduction of early weaning.

Transition constraints can facilitate or inhibit the achievement of a healthy transition, depending on the perception and mean- ing that an individual attributes to this experience. Therefore, identifying personal and environmental constraints is essential to understanding the achievement of a healthy transition. Personal constraints include neutral, positive, or negative meanings, as well as cultural beliefs and attitudes, socioeconomic status, and preparation and knowledge. Environmental constraints include community conditions, such as access to resources, support received from partners and family, and advice from respected sources, as well as the social conditions that surround them, such as social roles and cultural norms. Therefore, personal, community, or social conditions can facilitate or limit healthy transition processes and transition outcomes^(5)^.

Understanding the adaptations and facilitating and inhibit- ing factors present in the experience of these mothers will favor the transitional care promoted by nurses, making the changes part of a healthier and easier process for families. After a broad literature review, from the perspective of BF in CSHCN, it became evident that there is a lack of studies that comprehensively ad- dress this topic. Moreover, national and international research on the facilitators and inhibitors of BF in this population group is limited. In fact, new studies in this area are recommended in the literature^(7,8)^.

## OBJECTIVE

To describe the experiences of CSHCN mothers regarding BF in light of Meleis’s Transition Theory.

## METHODS

### Ethical aspects

The research followed the ethical guidelines of Resolution 466/2012 of the Brazilian National Health Council, and was ap- proved by the *Universidade Federal do Estado do Rio de Janeiro* Research Ethics Committee. Participants were assured of confi- dentiality, anonymity and freedom to withdraw from the research. Consent was formalized via an Informed Consent Form, with alphanumeric identification to guarantee anonymity.

### Study design

This is a qualitative, descriptive study, following the COn- solidated criteria for REporting Qualitative research (COREQ) standards^(9)^.

### Methodological procedures

Initially, the CSHCN Screener®, a questionnaire to identify and characterize CSHCN, translated and adapted for Brazil^(10)^, was applied to mothers waiting for care in the study setting. This instrument investigates five living and health conditions, with “yes” or “no” answers. Regardless of the stage of the question- naire, an affirmative answer indicates that a child is a CSHCN^(11)^. The purpose of this questionnaire was to identify the mothers of these children, considering that the study setting was a health- care service focused on screening and intervening in babies with potential developmental impairments. Therefore, there could be children who did not fit into the CSHCN category. Subsequently, the Knowledge Tree dynamic was carried out with mothers, a technique originating from the Creative and Sensitive Method (CSM), which is based on art-based research, Paulo Freire’s six core ideas, and the sharing of knowledge in the group meeting^(12)^. The chosen dynamic, based on the tree’s metaphorical language, allows us to explore the experiences lived by individuals when faced with a phenomenon, which allows each mother to associate the parts of the tree with their transitional process.

### Study setting

The study was conducted in an outpatient service of a follow- up program located in the city of Volta Redonda, in the southern region of the state of Rio de Janeiro, which focuses on monitoring high-risk newborns, seeking early diagnosis of developmental disorders, timely interventions and guidance for caregivers. Chil- dren referred to the service come from Neonatal Intensive Care Units (NICUs) and Primary Care in the city itself and in other cities in the southern region of the state of Rio de Janeiro.

### Data source

Twenty mothers of CSHCN participated in the study. The inclusion criteria were to be a mother (over 18 years old) of a CSHCN up to two years of age who was BF or had previously breastfed, identified using the CSHCN Screener®. The exclusion criterion was to be a mother of a CSHCN with physical or psy- chological impairment in their health status, according to the mother’s report, which required full care, thus making it difficult for them to participate in the study.

### Data collection and organization

Data production was conducted by the first author of the research, who was trained by the supervising professor, a PhD in nursing, with experience in research of this nature. Participants were approached individually for a brief presentation by the researcher and the objectives of the research while they waited for their children to be seen. There was no contact with partici- pants on previous days. Therefore, initially, in a private room, to ensure privacy, the CSHCN Screener® was applied. After a child was identified as CSHCN, data characterizing mothers and chil- dren (age, city of residence, BF time, type of pregnancy, reason for monitoring and care demands) were collected.

Nine dynamics were carried out in groups of three to four people, in which each mother participated in a single group. The dynamics occurred in five stages: 1) reception and presentation of the researcher and each member of the group and the question generating debate (QGD): visualizing your experiences related to BF as a tree, what was the process of breastfeeding your child with special health needs like?; 2) artistic production; 3) social- ization of group members, in which each one presented their artistic production; 4) critical reflection, consisting of collective analysis and discussion; 5) thematic synthesis, after discussion and recoding of subthemes from themes, in which the group itself validated the data.

The researcher wrote the QGD on a white card, which was stuck to the wall of the room where the dynamic took place. Each mother individually drew the “BF tree” on a card, using the following analogies: the roots represented “the reasons that led the mother to breastfeed her child with special health needs”; the trunk was associated with “adaptations and changes during the BF transition process”; in the treetop, there were fruits that symbolized “the facilitators and inhibitors that interfere with the BF of these children”. The dynamics lasted an average of 30 minutes. Data production took place on weekdays in March 2023, until theoretical data saturation was achieved^(13)^. It is noteworthy that all children were identified as CSHCN, only two mothers refused to participate in the research and there were no withdrawals.

### Data analysis

Mothers’ statements from the dynamics were transcribed in full. To form the text *corpus*, processed in the software *Interface de R pour les Analyses Multidimensionnelles de Textes et de Ques- tionnaires* (IRAMUTEQ), statements were grouped by dynamics, considering that the CSM is conducted in groups; therefore, the corpus was composed of nine texts from the nine dynamics. The textual data analysis occurred in three stages: 1) text *corpus* preparation and coding; 2) textual data processing in the software; and 3) interpretation of findings by the researchers.

Descending Hierarchical Classification (DHC) was adopted, a technique that processes data through cluster analysis, where text segments of a *corpus* are grouped based on the co-occur- rence of lexical forms, through calculations of distances and proximities with chi-square tests (χ2)(14,15). With the processed data, interpretation was carried out through Minayo’s thematic analysis^(16)^. Simultaneously, the search for the cores of meaning was conducted in light of Meleis’s Transitions Theory, providing a description, understanding, interpretation and explanation of the phenomenon investigated^(6)^.

## RESULTS

Twenty mothers from a total of 23 identified CSHCN partici- pated in the study. Mothers’ ages ranged from 18 to 45 years, with the majority (35.0%) between 31 and 35 years. Regarding the municipality, the majority (85.0%) lived in Volta Redonda, while 10.0% lived in Barra Mansa and 5.0% in Três Rios. Of the 23 children, 69.6% were from singleton pregnancies and 30.4% from twin pregnancies, and only 48.0% were being breastfed. The mean BF time was approximately four months. Care demands included modified usual care (100.0%), developmental care (65.3%) and medication (30.0%), with no reports of technological care. The origins of special health needs were classified based on the reasons for follow-up, highlighting prematurity (69.6%), re- spiratory distress (26.1%), sepsis (13.1%), hypoglycemia (13.1%),

bronchopulmonary dysplasia (8.7%), pulmonary atelectasis (8.7%), perinatal hypoxia (4.4%), placental abruption (4.4%), intrauterine growth restriction (4.4%), Down syndrome (4.4%), congenital heart disease (4.4%), developmental delay (4.4%), gastroesophageal reflux (4.4%), hyaline membrane disease (4.4%), esophageal atresia with tracheoesophageal fistula (4.4%), laryngotracheomalacia (4.4%), *Pectus Carinatum* (4.4%), congenital torticollis (4.4%), cardiac arrhythmia (4.4%) and brain alteration (4.4%).

**Figure 1:**
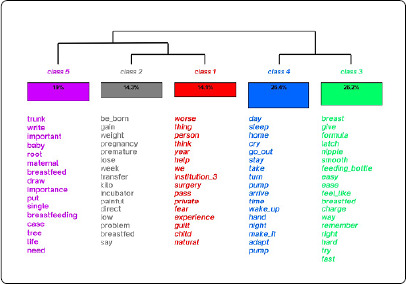
Dendrogram related to the experience of mothers of children with special health needs regarding breastfeeding. Volta Redonda, Rio de Janeiro, Brazil, 2024

The text *corpus*, after processing by IRAMUTEQ, consisted of a total of 16,003 word occurrences, of which 1,108 were distinct words and 466 had a single occurrence (hapax). In the DHC, five stable classes of text segments were formed, with a retention rate of 91.11% of the 461 text segments. The stemming resulted in 1,108 stems, with 916 active forms. The text *corpus* was divided into two independent chunks. The first consisted of class 3, rep- resented by the color green (26.19%), and class 4, represented by the color blue (26.43%). The second chunk was composed of class 5, represented by the color pink (19.05%), and an ad- ditional subdivision formed by class 1, represented by the color red (14.05%), and class 2, represented by the color gray (14.29%). The latter present more similar semantic contents, although with some differentiation (Figure 1).

After reading the associated terms and the text segments related to them, it was possible to assign names to the classes through the interpretative process of the core meanings generated by the speeches. This procedure provided a deeper understanding of the transitional process experienced by the mothers of CSHCN in relation to BF.

### Class 1 – Milk bank support and maternal emotional challenges

In class 1, some words related to facilitating and inhibiting conditions during the transitional BF process for these CSHCN mothers stood out. The milk bank was identified as a facilitator of this process, because without its assistance, BF would be even more challenging for these mothers. The support received with guidance on how to massage the breast to extract breast milk proved to be essential for their children to be able to receive milk, even if it was through an orogastric tube.

*I went to seek help, I went to donate milk at institution_3, and there I received a totally different service from the private network.* (M19)

*I also had help to extract milk at the milk bank, but I had to mas- sage mine.* (M7)

*Because I extracted it so I could give it to him through a tube.* (M13)

It was observed that staying in the NICU was an inhibiting factor in the transition process of these mothers. Having to leave their child in the hospital, away from their care, without being able to breastfeed, had an even greater impact on the emotional state of these women. Thus, some feelings experienced by mothers were identified, such as frustration at not being able to breastfeed immediately and leaving the hospital without their child, in addi- tion to the anxiety of waiting a whole night to stay with their child.

*We wait for the birth at the right time. We’ve already held the baby there, breastfed the baby there and left with our child. Now you leave them there, you make all the plans and leave them behind? That thing destroys you because your emotions go down.* (M14)

*When you leave, you have to leave them behind and you have to wait all night to see the next day and know how they’re going to be. It’s the worst thing.* (M20)

Therefore, the emotional aspect of these mothers also proved to be an inhibiting factor in the transition process. The feeling of fear permeated their experiences, whether due to the concern of not being able to produce enough milk or the fear that their child would present some change in their clinical condition dur- ing BF, due to the intense demands of care. Moreover, the fear of complications for the child while hospitalized, away from the mother’s presence, was also evidenced.

*You who have been through so much are you afraid, will the saturation drop? I spent the whole time looking at the monitor.* (M19)

*You go in all happy because you know you’re going to see it, but you go in scared because you don’t know what you’re going to see in the incubator, if she spent a night well, that’s the terror.* (M20)

### Class 2 – Maternal motivations for breastfeeding and challenges of staying in the Neonatal Intensive Care Unit

This class encompasses several terms, identifying the reasons that led mothers to breastfeed their children with special health needs as well as some of the transition conditions experienced by these women. The semantic content revealed that mothers in this study followed different paths in relation to BF, influenced by the prematurity of their children’s birth and their stay in intensive care.

The reasons that led mothers to breastfeed were diverse, highlighting the benefits of BF for the children’s well-being and development, in addition to strengthening the mother-child bond through contact during BF. Previous BF experience was also cited as a reason for wanting to experience BF.

*The issue of well-being, her development, in addition to the care, the contact between the mother and her child.* (M5)

*I have always liked breastfeeding because I have always breastfed all my children.* (M12)

The term“premature” was often cited as an inhibiting factor in the transition process, as experiencing prematurity is a challeng- ing experience. Even though mothers really want to breastfeed, they often do not feel prepared to deal with all the situations that arise when having a premature baby.

*I always dreamed about breastfeeding. I really wanted to breastfeed and I didn’t expect to have to go through the whole process that I went through, having a premature baby and having to have a feeding tube.* (M19)

*He was also born prematurely due to some situations, and I think that ended up causing this difficulty, because I couldn’t have peace of mind to have milk.* (M17)

Still in this context of prematurity, another inhibiting condition presented was the delay in producing breast milk. Thus, the lack of milk supply made the BF process even more challenging, since it took a few days for colostrum to be produced.

*I had difficulty producing milk, and the lack of milk supply. My birth was very premature, so I didn’t have milk. My milk didn’t come in.* (M16)

*When she was born, it took about five or six days for a little milk to come out and it had to harden a little. That was the difficulty.* (M20)

### Class 3 – Maternal adaptations, facilities and difficulties in breastfeeding

In this class, the use of a breast pump was highlighted, in the ex- perience of some participating mothers, as a facilitating factor, given that the pressure exerted, combined with the extraction of a little milk, allowed for areolar malleability, facilitating the child’s latch on.

*The pump made it much easier, because she would pull, apply pressure and then she would put his mouth there, he would breastfeed. It would come out easily for him.* (M2)

*I did it with the pump too. It made it easier because, after I put her on my breast and had to pick her up, make the teat and put it in her mouth, she would breastfeed easily.* (M3)

Another facilitating factor revealed by mothers was the ease with which the baby was latched on. Even though children had special care demands, compared to children of the same age, they demonstrated ease in BF. Another mother reported that the experience of witnessing the BF process of relatives contributed positively to her transition process.

*So, it was easy for them to latch on very quickly. They sucked very quickly. They learned to suck very quickly.* (M1)

*They latched on to the breast perfectly. The easy part was that she latched on well.* (M3)

*It was easy, as I had already seen my cousins who normally breastfed, so it was easy, I already knew how to latch on, so it was easy.* (M11)

On the other hand, formula and/or bottle feeding were identi- fied as inhibiting conditions in the transition to BF for CSHCN, especially due to society’s judgment and self-pressure for not BF.

*The difficulty for me was other people’s opinions, because you have to breastfeed. Only those who are with me all the time know how much I’m trying to breastfeed her, so I don’t accept others saying that I’m not trying to breastfeed her.* (M20)

*Just like I was, in the beginning, I was very demanding of myself, I was like, “My son, take the breast, take the breast, take the breast”. I cried.* (M13)

### Class 4 – Challenges and adaptations to breastfeeding during hospitalization in the Neonatal Intensive Care Unit

In class 4, the daily commute from home to the hospital in the hope of experiencing the process of BF their child emerged as yet another adaptation during this transition. Mothers reported that they went every day to express breast milk, since their children did not yet have the strength to suckle.

*I had to leave the house every day to breastfeed them and see them. It was a change, because I had to leave the house every day to be with them.* (M1)

*I’m going to see him every day, every day to pump milk so he can breastfeed, every day to see him there, I had to adapt a lot to that.* (M2)

Another challenge they experienced was spending the entire day in intensive care to make the most of their child’s presence. In this context, understanding that their child needed to breastfeed several times a day and in small quantities, paying attention to feeding times, and even waking them up to breastfeed were changes they learned throughout the transitional experience.

*I would arrive early and spend the day there to make the most of it. I would go to work and sometimes I would get home at 11:00 p.m.* (M1)

*So, I had to be careful to know the exact time for him to breast- feed because there were days when he didn’t cry. He would sleep beautifully.* (M7)

*Today, I understand that it’s little by little, even if it’s several times a day.* (M8)

Participant M4 even mentioned the need to adapt to stimuli to wake her daughter up, such as tickling her foot and removing the child’s clothes so that she would feel cold and thus wake up to be able to breastfeed.

*So, stimulating her, waking her up, tickling her foot, taking off her clothes, letting her feel cold, because she always slept, picked her up and slept and I stayed, sometimes for an hour, even disrupting the hospital routine so she could breastfeed.* (M4)

The difficulty in sleeping at home while her son remained hospitalized in intensive care was also cited as a hindering factor in the experience. Returning home without her child was pointed out as a challenging situation, since the mother continued to pro- duce breast milk, but did not have her son nearby to breastfeed, which made the transition painful for her. The feeling of frustra- tion appeared at different moments in the transition process.

*When I started sleeping there, everyone goes to sleep, but not me. I’m going to stay here. I sleep better here than at home, because I didn’t sleep at home.* (M20)

*The hard part was getting home and my breasts hurting and not having him to breastfeed.* (M12)

*I cried a lot. I went home really frustrated because it was just like she said. He just had to put his mouth on it and he would breastfeed, but it wasn’t like that.* (M13)

### Class 5 - Knowledge Tree dynamics and maternal reasons for breastfeeding

This class presented many active words referring to the dy- namics used in the study, such as “trunk”, “write”, “root”, “draw”, “place”, “tree”, “pen” and “fruit”. The recurrence of these terms in the textual corpus confirms the effectiveness of the Knowledge Tree dynamics for data production. Most participants revealed that the desire to breastfeed was due to the importance of BF for the health of babies, immunity, growth and development, espe- cially for the neurological system, as well as for their immunity.

*I wrote that I really wanted to breastfeed because it is very important for the health of babies, for their neurological system. It is very important for their health and immunity.* (M1)

*The root is the issue of growth, because breast milk is important.* (M11)

*In the trunk, the changes I had were that I had to do a lot of stimulation.* (M15)

The importance of nutrients present in milk and the benefits of breastfeeding for the healthy growth of the child were also reasons why mothers wanted to breastfeed their children. It is worth noting that creating a bond between mother and child during BF appears as another reason for wanting to breastfeed. Participants also pointed out the desire to be the best mothers for their children. However, they mentioned that the fact of not exclusively BF does not make them any less of a mother.

*The reason I wanted to start breastfeeding was because I was always aware of the importance of breast milk, which is the nutrients my baby needs. It is good for creating a bond between mother and child, which is what led me to want to breastfeed.* (M14)

*I always wanted to breastfeed, I always did. I think it is a very beautiful bond between mother and child, I think it is when you know for sure that you are a mother.* (M17)

*Not breastfeeding itself, but the pressure on myself, of wanting to be a mother, because I am not being less of a mother because I am not breastfeeding completely, but unfortunately this gets in the way of our minds.* (M14)

## DISCUSSION

The results of this research provided an in-depth understand- ing of the transitional process faced by CSHCN mothers during BF. The experience of staying in the hospital became a crucial element for these mothers, requiring significant adaptations in their daily routine. Infant hospitalization causes drastic changes in family dynamics, resulting in a different experience than what families are used to^(17)^. The need for hospitalization has differ- ent repercussions for the child’s life and, mainly, for their family members, as indicated in the literature, especially considering the bond created between mother and child during pregnancy, childbirth and postpartum^(18)^.

Mothers’ commitment to BF their child with special health needs was motivated both by a genuine desire to provide the best care and by knowledge of the benefits of BF and/or by the child’s fragile health condition. These findings support research in which mothers, even faced with the difficulties of BF a newborn who underwent surgery, demonstrated the desire to breastfeed or provide only their own milk to their child^(19)^.

The BF process is complex due to the physical, psychological and emotional peculiarities of mothers. This transition becomes even more challenging given the newborn’s physiological and neurological immaturity, such as prematurity. Furthermore, hospitalization itself generated daily concerns and tensions for families, since most were anxiously waiting to see their children again, in addition to obtaining information about the newborn’s health and BF status. This situation led family members to go to the hospital frequently to minimize their anxieties, which is in line with the literature(20,21), demonstrating the commitment to overcoming the challenges of BF a CSHCN.

During the transitional process, it was important to identify and describe the changes to better understand it^(5)^. Based on partici- pants’ experiences, the changes observed during the transition process included frequent visits to the hospital, delay in starting BF, practice of expressing breast milk, BF children with special needs, and adaptation to scheduled feeding times. Staying in the hospital full-time, without taking turns, also caused physical fatigue and emotional exhaustion. However, mothers preferred to deal with this exhaustion in order to stay close to their children.

In a study in Campo Grande, Brazil, participants expressed a desire to be at home, but spent the day in the hospital, intensifying stress and physical and psychological exhaustion. The lack of alternatives for taking turns and the hesitation to leave their child in the care of others caused some mothers to remain in the NICU all day^(22)^, similar to what was observed in this study. Mothers also considered it part of their maternal role to be with their child during hospitalization, believing that their presence would speed up the newborn’s recovery.

The results highlighted the importance of support from milk bank professionals for mothers. Another study also indicated that supportive behaviors, massage and milk extraction techniques, guid- ance and observation of BF, together with support and assistance in the transition process, were crucial to the experience of mothers with children admitted to the NICU^(19)^. The results highlighted the importance of support from milk bank professionals for mothers. Another study also indicated that supportive behaviors, massage and milk extraction techniques, guidance and observation of BF, together with support and assistance in the transition process, were crucial to the experience of mothers with children admitted to the NICU^(23)^.

Although milking is not the practice of BF itself, mothers and professionals consider that milk extraction constitutes one of the stages of BF, as it allows the obtaining of breast milk, either to be offered directly to the baby or processed in the milk bank for later use^(24)^. In this study, mothers also emphasized the relevance of expressing breast milk to feed their children with their own milk while they were unable to breastfeed directly.

Mothers of premature newborns have adopted different care practices for their babies, considering them to be more fragile and delicate than full-term babies. Research indicates that, due to the immaturity of babies, these mothers need to follow specific feeding schedules. Since premature newborns do not express clear signs of hunger, mothers need to respect the intervals, offering BF every three hours to avoid complications(25,26). This evidence corroborates the findings of this study, in which moth- ers adapted to the short intervals between feedings and often needed to stimulate babies to wake them up.

Given the unexpected changes and challenges of the transi- tion process, some mothers realized that BF, which should be special and pleasurable, was proving to be too challenging for their babies. As a result, they chose to stop BF, despite being aware of the many benefits of BF. The success or failure of BF depends on several factors, without a single determining factor. BF under duress, without the mother feeling comfortable, can be psychologically harmful to both her and her child. BF should not be a painful obligation, but rather an opportunity to create an intimate bond and strengthen emotional ties^(27)^.

The results suggest that the transition process from BF to BF for CSHCN mothers was not healthy for all participants. At the time of data collection, only 48% of children were being breastfed. The unexpected changes negatively impacted the transition. Therefore, healthcare professionals, especially nurses, should be aware when negative aspects predominate over positive ones, intervening to minimize emotional distress and promote a smoother and better-quality transition for mother and child.

According to another study, the milk bank played a crucial role in encouraging BF among mothers who had been in the NICU, providing guidance and support at times of great vulner- ability for women, due to the frustration of a different birth than expected, experiencing the postpartum period away from their child, and difficulties in BF. Moreover, the milk bank was essential for understanding BF and its management. Professionals reassured mothers about milk production, provided guidance on how to stimulate and massage their breasts, express breast milk, and shared information on the correct latch to avoid cracked nipples^(28)^.

Given the complexity of the transition to BF for CSHCN, mothers were found to be demanding of themselves in relation to BF. Nurses should be aware of the dimensions of maternal psychological dis- tress in order to intervene empathetically, prioritizing the general well-being of the mother-child dyad. BF should not be an imposed obligation, but rather an act that strengthens the emotional bond^(27)^. However, watching their child use technology to feed themselves, combined with the impossibility of BF, exposes the mother to an unexpected reality. The means by which the newborn feeds becomes an external mechanism, not originating from her. This experience can cause suffering, frustration and even guilt in the mother, especially for those who see BF as a fundamental part of the maternal role^(23)^. In this context, mothers become self-demanding and responsible for providing sustenance for their children, whether due to the hope created in BF the imaginary baby idealized during pregnancy or due to prior knowledge of the benefits of BF, especially when faced with a real baby who needs special care.

Within the conditions of the transition, the stigmas present in society acted as inhibitors of the experience^(5)^. Social judgment of mothers of children with special needs who are unable to breastfeed or who need supplementary feeding was an inhibit- ing factor in the transition, justified by the responsibility for having to breastfeed according to the opinion of people in the socio-family context. In view of this, nurses must know the social and community environment in which the mother-child dyad is inserted to help deconstruct these judgments^(24)^.

The analysis of mothers’ experience was favored by the Afaf Meleis theoretical framework^(29)^, which helps to understand the importance of transitional care for nurses, contributing to personal growth and maturity, seeking balance and stability in the lived experience. Thus, some nursing interventions are suggested, such as: welcoming, offering humanized and comprehensive care; sharing guidance on the importance of BF and child care, including adaptations in view of the different care demands; providing emotional support; reducing anxiety and pressure regarding BF; reassuring about breast milk production; explaining the importance of using infant formula when necessary; promot- ing awareness that BF is positive and healthy for mothers and children; actively listen; and support mothers when they decide to stop or give up BF.

### Study limitations

This study was conducted in a single healthcare service in the countryside of the state of Rio de Janeiro, using exclusively the CSM Knowledge Tree dynamics with mothers of CSHCN as a data source. This specific approach limits the more comprehensive generalization of the findings. Therefore, the need to develop new research on the subject is highlighted, including CSHCN and their families, as well as healthcare professionals, especially nurses.

### Contributions to nursing, health or public policy

The findings contribute to supporting nurses’ transitional care, in order to facilitate the transition process of BF CSHCN, in order to guarantee BF practice and continuity with autonomy, confidence and quality as the difficulties and negative feelings generated by transition are reduced. Furthermore, the application of Meley’s Transition Theory contributes to the advancement of future periodic productions based on this nursing theory and to the development of nursing care models aimed at families experiencing transitional experiences of BF CSHCN.

## FINAL CONSIDERATIONS

The study findings revealed the adaptations and the facilitat- ing and inhibiting conditions present in the experience of CSHCN mothers in the transitional process of BF. Thus, it was found that participants experienced multiple, complex and simultaneous transitions, covering several aspects related to the child’s birth, prematurity, hospitalization, special health needs, in addition to the constant demands of hospital environments and the BF process itself, which is sometimes different from other children.

Many faced emotional, physical and social challenges when trying to breastfeed their children in the midst of so many adverse circumstances. This reinforces the importance of joint action between nurses and other healthcare professionals with mothers for quality transitional care in CSHCN BF, in order to support and offer support, including if a mother decides to interrupt and/or give up this process (when the hindering conditions outweigh the facilitating ones).

## Data Availability

Not applicable
