# Supplementary material for: Transitional experiences of mothers of children with special health needs in breastfeeding
Source: Rev Bras Enferm. 2025 Aug 25;78(Suppl 2):e20240272. doi: 10.1590/0034-7167-2024-0272 (PMC12380418; doi:10.1590/0034-7167-2024-0272)
Supplement: Translation

# Translation: Experiências transicionais de mães de crianças com necessidades especiais de saúde no aleitamento materno

**DOI:** 10.1590/0034-7167-2024-0272pt

**Authors:** Ana Carla Silveira de Sá, Fernanda Garcia Bezerra Góes, Andressa Neto Souza, Laura Johanson da Silva, Aline Cerqueira Santos Santana da Silva, Juliana Rezende Montenegro Medeiros de Moraes, Liliane Faria da Silva, Fernando Rocha Porto

**Affiliations:** I Universidade Federal do Estado do Rio de Janeiro. Rio de Janeiro, Rio de Janeiro, Brazil.; II Universidade Federal Fluminense. Rio das Ostras, Rio de Janeiro, Brazil.; III Universidade Federal do Rio de Janeiro. Rio de Janeiro, Rio de Janeiro, Brazil.; IV Universidade Federal Fluminense. Rio de Janeiro, Rio de Janeiro, Brazil.

**Keywords:** Criança, Crianças com Deficiência, Mães, Aleitamento Materno, Doença Crônica

## Abstract

**Objetivo:**

descrever as experiências de mães de crianças com necessidades especiais de saúde quanto ao aleitamento materno à luz da Teoria das Transições de Meleis.

**Métodos:**

estudo qualitativo, mediante nove dinâmicas da Árvore do Conhecimento do Método Criativo Sensível, com 20 mães, em março de 2023. Dados foram processados no *software Interface de R pour les Analyses Multidimensionnelles de Textes et de Questionnaires* e submetidos à análise temática.

**Resultados:**

as mães vivenciaram transições múltiplas, complexas e simultâneas, abrangendo aspectos relacionados à prematuridade e à hospitalização na terapia intensiva. Muitas enfrentaram desafios emocionais, físicos e sociais ao tentar amamentar seus filhos diante das circunstâncias adversas. Um facilitador foi o apoio dos profissionais de saúde do banco de leite.

**Considerações finais:**

é relevante a atuação conjunta entre enfermeiros e demais profissionais de saúde junto às mães para o cuidado transicional de qualidade no aleitamento materno de crianças com necessidades especiais de saúde.

## INTRODUÇÃO

A taxa global de aleitamento materno exclusivo (AME) nos primeiros quatro meses de vida é de 35%, e no Brasil, a média de AME é de apenas 23 dias^(1)^. O Estudo Brasileiro de Alimentação e Nutrição Infantil revelou que, entre fevereiro de 2019 e março de 2020, a prevalência de aleitamento materno (AM) na primeira hora de vida foi de 62,4%, com maior prevalência na região Norte (73,5%), seguida das regiões Centro-Oeste (64,0%) e Nordeste (63,2%), enquanto as regiões Sul (61,8%) e Sudeste (58,5%) apresentaram as menores prevalências. Ademais, a prevalência de AME em menores de 6 meses foi de 45,8%, também aquém das recomendações internacionais^(2)^.

A amamentação de crianças com necessidades especiais de saúde (CRIANES) tende a ser ainda mais breve em comparação com crianças saudáveis devido às diferentes demandas de cuidado, o que contribui para o desmame precoce. Por exemplo, crianças com síndrome de Down são amamentadas com menos frequên- cia do que aquelas sem a síndrome^(3)^. Além disso, a manutenção do AM é mais desafiadora em recém-nascidos prematuros e de risco do que em recém-nascidos a termo e saudáveis, devido a fatores como patologia de base, fragilidade clínica, hospitalização prolongada, separação entre mãe e bebê, e estresse materno^(4)^. O nascimento de uma criança e o AM são eventos de transição marcados por diversas mudanças na vida das pessoas envolvidas, com implicações e adaptações distintas para mãe, bebê e família. A transição da mãe para o aleitamento envolve a transformação do seu corpo, que se inicia desde a gestação. O corpo, antes ex- clusivamente seu, torna-se uma fonte de alimento para a criança. Portanto, essa transição implica passar de um estado (lugar ou condição) estável para outro, exigindo que elas incorporem novos conhecimentos, alterem seus comportamentos e mudem a defi- nição do *self* (identidade). A transição é saudável quando ocorre o domínio de novas habilidades e comportamentos necessários para gerenciar a nova situação de vida, bem como a construção de uma nova identidade^(5)^.

A complexidade de todas essas transições se amplifica quando as mães vivenciam a experiência de ter um filho com necessidade especial de saúde e de amamentá-lo. Nesse contexto, é crucial compreender as transições em termos de sua natureza, tempo- ralidade, gravidade e expectativas pessoais, familiares e sociais^(5)^. A Teoria das Transições^(6)^ surge como uma base teórica essencial para aprofundar a análise do processo transicional vivenciado por mães de CRIANES durante o AM. Essa compreensão é fundamental para orientar o cuidado transicional do enfermeiro, beneficiando uma transição saudável para essa população. Ao oferecer suporte baseado nas reais necessidades e interesses dos envolvidos, esse profissional atua na promoção da autonomia e do bem-estar, contribuindo para a redução do desmame precoce.

Os condicionantes da transição podem facilitar ou inibir o alcance de uma transição saudável, dependendo da percepção e do significado que o indivíduo atribui a essa experiência. Logo, identificar os condicionantes pessoais e ambientais é fundamental para entender o alcance da transição saudável. Os condicionantes pessoais incluem significados neutros, positivos ou negativos, além de crenças e atitudes culturais, *status* socioeconômico, e preparação e conhecimento. Os condicionantes ambientais abrangem as condições comunitárias, como o acesso a recursos, o apoio recebido por parceiros e familiares e os conselhos de fon- tes respeitadas, assim como as condições sociais que envolvem, como os papéis sociais e as normas culturais. Portanto, condições pessoais, comunitárias ou sociais podem facilitar ou limitar os processos de transições saudáveis e os resultados das transições^(5)^. Conhecer as adaptações e os condicionantes facilitadores e inibidores presentes na experiência dessas mães favorecerá o cuidado transicional promovido por enfermeiros, tornando as mudanças parte de um processo mais saudável e leve para as famílias. Após ampla revisão da literatura, na perspectiva do AM de CRIANES, tornou-se evidente a escassez de estudos que abordem de forma abrangente essa temática. Além disso, são limitadas as pesquisas nacionais e internacionais sobre os facilitadores e inibidores do AM nesse grupo populacional. Inclusive, a realização novos estudos nessa vertente é recomendada na literatura^(7,8)^.

## OBJETIVO

Descrever as experiências de mães de CRIANES quanto ao AM à luz da Teoria das Transições de Meleis.

## MÉTODOS

### Aspectos éticos

A pesquisa seguiu as diretrizes éticas da Resolução n° 466/2012 do Conselho Nacional de Saúde, e foi aprovada pelo Comitê de Ética em Pesquisa da Universidade Federal do Estado do Rio de Janeiro. As participantes foram asseguradas do sigilo, anonimato e liberdade de desistir da pesquisa. O consentimento foi for- malizado via Termo de Consentimento Livre e Esclarecido, com identificação alfanumérica para garantir anonimato.

### Tipo de estudo

Estudo de abordagem qualitativa, do tipo descritivo, seguindo as normativas do *COnsolidated criteria for REporting Qualitative research* (COREQ)^(9)^.

### Procedimentos metodológicos

Inicialmente, aplicou-se o CSHCN *Screener*®, um questio- nário para identificar e caracterizar as CRIANES, traduzido e adaptado para o Brasil^(10)^, com as mães que aguardavam pelo atendimento no cenário de estudo. Este instrumento investiga cinco condições de vida e saúde, com respostas do tipo “sim” ou “não”. Independentemente da etapa do questionário, uma resposta afirmativa indica que a criança é uma CRIANES^(11)^. A aplicação deste questionário visou identificar quais eram as mães dessas crianças, considerando que o cenário do estudo foi um serviço de saúde voltado ao rastreamento e à intervenção em bebês com potencial comprometimento no desenvolvimento. Dessa forma, poderia haver crianças que não se enquadrassem na categoria de CRIANES. Posteriormente, foi realizada com as mães a dinâmica da Árvore do Conhecimento, uma técnica originada do Método Criativo e Sensível (MCS), que tem como base a pesquisa baseada em arte, as seis ideias forças de Paulo Freire e o compartilhamento de saberes no encontro grupal^(12)^. A dinâmica escolhida, fundamentada na linguagem metafórica da árvore, permite explorar as experiências vivenciadas pelo indivíduo diante de um fenômeno, o que possibilita que cada mãe associe as partes da árvore ao seu processo transicional.

### Cenário de estudo

O estudo foi conduzido em serviço ambulatorial de um progra- ma de *follow-up*, localizado no município de Volta Redonda, na região Sul Fluminense do estado do Rio de Janeiro, que concentra esforços no acompanhamento de recém-nascidos de alto risco, buscando diagnósticos precoces de distúrbios de desenvolvi- mento, intervenções oportunas e orientação aos responsáveis. Crianças encaminhadas para o serviço provêm de Unidades de Terapia Intensiva Neonatal (UTINs) e da Atenção Básica da própria cidade e de outros municípios da região Sul fluminense.

### Fonte de dados

Participaram do estudo 20 mães de CRIANES, cujos critérios de inclusão foram ser mãe (maior de 18 anos) de CRIANES com faixa etária de até dois anos de idade e que estivesse amamentando ou que já tivesse amamentado ao seio, identificadas por meio CSHCN *Screener*®. O critério de exclusão foi ser mãe de CRIANES com comprometimento físico ou psíquico em seu estado de saúde, de acordo com o relato da mãe, que exigisse atenção integral, dificultando, assim, sua participação no estudo.

### Coleta e organização dos dados

A produção de dados foi conduzida pela primeira autora da pesquisa, a qual foi treinada pela professora orientadora, doutora em enfermagem, com experiência em pesquisas dessa natureza. As participantes foram abordadas individualmente para uma breve apresentação da pesquisadora e dos objetivos da pesquisa enquanto aguardavam o atendimento de seus filhos. Não houve contato em dias anteriores com as participantes. Assim, inicialmente, em uma sala reservada, para garantir a privacidade, foi aplicado o CSHCN *Screener*®. Após a identificação da criança como CRIANES, foram coletados dados de caracterização das mães e das crianças (idade, município de residência, tempo de amamentação, tipo de gestação, motivo de acompanhamento e demandas de cuidado).

Foram realizadas nove dinâmicas em grupos de três a quatro pessoas, em que cada mãe participou de um único grupo. As dinâmicas ocorreram em cinco etapas: 1) acolhimento e apre- sentação da pesquisadora e de cada integrante do grupo e da questão geradora de debate (QGD): visualizando suas experiências relacionadas ao AM como uma árvore, como foi o processo de amamentar o seu filho com necessidade especial de saúde?; 2) produção artística; 3) socialização dos componentes do grupo, em que cada um apresentou a sua produção artística; 4) reflexão crítica, composta pela análise e discussão coletiva; 5) síntese temática, após a discussão e recodificação de subtemas a partir dos temas, em que o próprio grupo validou os dados.

A pesquisadora escreveu a QGD em uma cartolina branca, que foi colada na parede da sala onde ocorreu a dinâmica. Cada mãe desenhou, individualmente, em uma cartolina a “árvo- re-amamentação”, utilizando as seguintes analogias: as raízes representaram “as razões que levaram a mãe a amamentar seu filho com necessidades especiais de saúde”; o tronco foi associa- do às “adaptações e mudanças durante o processo de transição de amamentação”; na copa da árvore, estavam os frutos que simbolizavam“os facilitadores e os inibidores que interferem no AM dessas crianças”. As dinâmicas tiveram duração média de 30 minutos. A produção de dados ocorreu em dias úteis no mês de março de 2023, até a saturação teórica dos dados^(13)^. Ressalta-se que todas as crianças foram identificadas como CRIANES, apenas duas mães se recusaram em participar da pesquisa e não houve desistências.

### Análise de dados

As falas das mães oriundas das dinâmicas forram trans- critas na íntegra. Para formar o *corpus* textual, processado no *software Interface de R pour les Analyses Multidimensionnelles de Textes et de Questionnaires* (IRAMUTEQ), as falas foram agrupadas por dinâmica, considerando que o MCS é conduzido em grupo; logo, o *corpus* foi composto por nove textos das nove dinâmi- cas. A análise dos dados textuais ocorreu em três momentos: 1) preparação e codificação do *corpus* textual; 2) processamento dos dados textuais no *software*; e 3) interpretação dos achados pelos pesquisadores.

Adotou-se a Classificação Hierárquica Descendente (CHD), uma técnica que processa dados por meio de uma análise de agrupamentos (*clusters*), em que os segmentos de texto de um *corpus* são agrupados com base na coocorrência de formas lexicais, através de cálculos de distanciamentos e proximidades com testes qui-quadrado (χ2)(14,15). Com os dados processados, realizou-se a interpretação por meio da análise temática de Minayo^(16)^. Simultaneamente, a busca pelos núcleos de sentido foi conduzida à luz da Teoria de Transições de Meleis, proporcio- nando uma descrição, compreensão, interpretação e explicação do fenômeno investigado^(6)^.

## RESULTADOS

Participaram do estudo 20 mães de um total de 23 CRIANES identificadas. A faixa etária das mães variou de 18 a 45 anos, com a maioria (35,0%) entre 31 e 35 anos. Quanto ao município, a maioria (85,0%) reside em Volta Redonda, enquanto 10,0%, em Barra Mansa, e 5,0%, em Três Rios. Das 23 crianças, 69,6% são de gestações únicas e, 30,4%, de gestações gemelares, sendo que apenas 48,0% estavam sendo amamentadas. O tempo médio de amamentação foi de aproximadamente quatro meses. As demandas de cuidados incluíram cuidado habitual modificado (100,0%), de desenvolvimento (65,3%) e medicamentoso (30,0%), sem relatos de cuidado tecnológico.

As origens das necessidades especiais de saúde foram classifi- cadas com base nos motivos de acompanhamento, destacando-se a prematuridade (69,6%), desconforto respiratório (26,1%), sepse (13,1%), hipoglicemia (13,1%), broncodisplasia pulmonar (8,7%), atelectasia pulmonar (8,7%), hipóxia perinatal (4,4%), desco- lamento da placenta (4,4%), crescimento intrauterino restrito (4,4%), síndrome de Down (4,4%), cardiopatia congênita (4,4%), atraso no desenvolvimento (4,4%), refluxo gastroesofágico (4,4%), doença na membrana hialina (4,4%), atresia de esôfago com fístula traqueoesofágica (4,4%), laringotraqueomalácia (4,4%), *Pectus Carinatum* (4,4%), torcicolo congênito (4,4%), arritmia cardíaca (4,4%) e alteração cerebral (4,4%).

O *corpus* textual, após o processamento pelo IRAMUTEQ, cons- tituiu-se de um total de 16.003 ocorrências de palavras, sendo 1.108 palavras distintas e 466 com uma única ocorrência (hápax). Na CHD, formaram-se cinco classes estáveis de segmentos de texto, com um aproveitamento de 91,11% de retenção dos 461 segmentos de texto. A lematização resultou em 1.108 lemas, com 916 formas ativas. O *corpus* textual foi dividido em dois blocos independentes. O primeiro consistiu na classe 3, representada pela cor verde (26,19%), e na classe 4, representada pela cor azul (26,43%). O segundo bloco foi composto pela classe 5, repre- sentada pela cor rosa (19,05%), e por uma subdivisão adicional formada pela classe 1, representada pela cor vermelha (14,05%), e pela classe 2, representada pela cor cinza (14,29%). Essas últimas apresentam conteúdos semânticos mais semelhantes, porém com certa diferenciação (Figura 1).

**Figura 1:** Dendrograma relacionado à experiência de mães de crianças com necessidades especiais de saúde quanto ao aleitamento materno. Volta Redonda, Rio de Janeiro, Brasil, 2024

Após a leitura dos termos associados e dos segmentos de texto relacionados a eles, foi possível atribuir denominações às classes por meio do processo interpretativo dos núcleos de sentido gerados pelas falas. Esse procedimento proporcionou uma compreensão mais aprofundada do processo transicional vivenciado pelas mães de CRIANES em relação ao AM.

### Classe 1 – Apoio do banco de leite e desafios emocionais maternos

Na classe 1, destacaram-se alguns vocábulos relacionados aos condicionantes facilitadores e inibidores durante o processo transicional de amamentação para essas mães de CRIANES. O banco de leite foi identificado como um facilitador desse processo, pois, sem sua assistência, amamentar seria ainda mais desafiador para essas mães. O apoio recebido com orientações sobre como massagear o peito para retirar o leite materno se mostrou essencial para que seus filhos pudessem receber o leite, mesmo que fosse por meio de sonda orogástrica.

*Eu fui procurar ajuda, eu fui doar o leite na instituição_3, e lá eu recebi um atendimento totalmente diferente da rede privado.* (M19)

*Eu também tive ajuda para tirar o leite no banco de leite também, só que o meu eu fazia massagem.* (M7)

*Porque eu tirava para poder dar na sonda para ele.* (M13)

Observou-se que a permanência na UTIN foi um fator inibidor no processo de transição dessas mães. Ter que deixar o filho no hospital, longe do seu cuidado, sem conseguir amamentá-lo, impactou ainda mais o estado emocional dessas mulheres. Iden- tificam-se, assim, alguns sentimentos vivenciados pelas mães, como frustração por não poder amamentar imediatamente e sair do hospital sem o filho, além da ansiedade de esperar uma noite inteira para permanecer junto do filho.

*A gente espera nascer no tempo certinho. Já pegou ali, já amamentou ali e foi embora com seu filho. Agora você deixar ali, você fazer todos os planos e deixar para trás? Aquilo ali acaba com você porque seu emocional vai lá embaixo.* (M14)

*Na hora que você vai embora, tem que deixar elas para trás e você tem que esperar uma noite toda para você ver no outro dia e saber como que vai estar. É a pior coisa.* (M20)

Portanto, o aspecto emocional dessas mães também se mos- trou como um condicionante inibidor do processo transicional. O sentimento de medo permeou suas experiências, seja pela preocupação de não conseguir uma boa produção de leite ou pelo receio de que o filho apresentasse alguma alteração no quadro clínico durante o ato de amamentar, devido às intensas demandas de cuidado. Além disso, o medo de complicações para o filho enquanto estava internado, longe da presença materna, também foi evidenciado.

*Você que já passou por tanta coisa você tem medo, será que a saturação vai cair? Eu ficava o tempo inteiro olhando para o monitor.* (M19)

*Você vai toda feliz porque você sabe que vai ver, mas você entra com medo porque não sabe o que vai ver na incubadora, se ela passou uma noite bem, esse é o terror.* (M20)

### Classe 2 – Motivações maternas para amamentar e desafios da permanência na Unidade de Terapia Intensiva Neonatal

Esta classe abrange diversos termos, identificando as razões que levaram as mães a amamentar seus filhos com necessidades especiais de saúde, assim como alguns condicionantes da transição vivenciados por essas mulheres. O conteúdo semântico revelou que as mães deste estudo seguiram caminhos distintos em relação ao AM, influenciadas pela prematuridade do nascimento de seus filhos e pela permanência na terapia intensiva.

As razões que levaram as mães a amamentarem foram diversas, destacando os benefícios do AM para o bem-estar e desenvol- vimento da criança, além do fortalecimento do vínculo mãe e filho através do contato durante a amamentação. A experiência anterior de amamentar também foi citada como um motivo para desejar vivenciar o AM.

*A questão de bem-estar, o desenvolvimento dela, fora o acolhimento, o contato da mãe com o filho.* (M5)

*Sempre gostei de amamentar porque eu sempre amamentei todos os meus filhos.* (M12)

O termo “prematuro” foi frequentemente citado como um condicionante inibidor do processo de transição, pois vivenciar a prematuridade é uma experiência desafiadora. Mesmo que as mães desejem muito amamentar, elas muitas vezes não se sen- tiram preparadas para lidar com todas as situações que surgem ao ter um filho prematuro.

*Sempre sonhei o processo da amamentação. Quis muito amamentar e não esperava ter todo o processo que eu passei, de ter prematuro e ter que ficar com sonda*. (M19)

*Ele nasceu prematuro também por conta de algumas situações, e eu acho que isso acabou gerando essa dificuldade, porque eu não consegui ter paz para ter leite.* (M17)

Ainda neste contexto da prematuridade, outro condicionante inibidor apresentado foi a demora em produzir o leite materno. Assim, a falta de apojadura tornou o processo de amamentação ainda mais desafiador, já que levou alguns dias para a produção do colostro.

*Eu tive dificuldade de produzir leite, e a falta de apojadura. Meu parto foi muito prematuro, então eu não tive leite. O descer do leite não aconteceu comigo*. (M16)

*Quando ela nasceu, demorou uns cinco, seis dias para sair um pouquinho de leite e teve que endurecer um pouquinho. A difi- culdade foi essa.* (M20)

### Classe 3 – Adaptações, facilidades e dificuldades maternas para amamentar

Nesta classe, o uso da bomba extratora de leite foi aponta- do, na experiência de algumas mães participantes, como um condicionante facilitador, dado que a pressão exercida somada a extração de um pouco de leite, permitia uma maleabilidade areolar, facilitando a pega da criança.

*A bomba facilitou bastante, porque ela já puxava, dava uma pressão e depois ela colocava a boca dele ali, ele mamava. Já saia com facilidade para ele.* (M2)

*Fazia com a bombinha também. Facilitou porque, depois que colocava ela no peito e tinha que pegar e fazer o bico e botar na boca dela, ela mamava tranquilo.* (M3)

Outro condicionante facilitador revelado pelas mães foi a facili- dade com a pega. Mesmo as crianças apresentando demandas decuidados especiais, comparada às crianças da mesma idade, elas demonstraram facilidade para mamar no peito. Outra mãe relatou que a experiência de presenciar o processo de amamentar de pa- rentes contribuiu positivamente para o seu processo transicional.

*Então, facilidade foi que eles pegaram o peito bem rápido. Sugaram muito rápido, aprenderam a sugar muito rápido.* (M1)

*Pegava direitinho o peito. A facilidade é que ela pegou bem o peito.* (M3)

*Facilidade, como eu já estava vendo os meus primos que normal- mente dava mama, então, foi tranquilo, eu já sabia como tinha que pegar, então foi tranquilo.* (M11)

Por outro lado, a fórmula e/ou a mamadeira foram identifica- das como condicionantes inibidores na transição de amamentar as CRIANES, especialmente pelo julgamento da sociedade e da própria autocobrança por não estar amamentando.

*A dificuldade foi, para mim, as opiniões das pessoas, porque você tem que dar peito. Só quem está comigo o tempo todo sabe como eu estou tentando dar mama para ela, então, eu não aceito os outros falarem que eu não estou tentando dar mama para ela*. (M20)

*Igual eu ficava, no começo, eu me cobrava muito eu ficava com ele vai meu filho pega o peito, pega o peito, pega o peito. Eu chorava.* (M13)

### Classe 4 – Desafios e adaptações para amamentar durante a internação na Unidade de Terapia Intensiva Neonatal

Na classe 4, o deslocamento diário da casa ao hospital na expectativa de vivenciar o processo de amamentar o seu filho surgiu como mais uma adaptação no decorrer desta transição. As mães relataram que iam todos os dias para tirar o leite materno, visto que as crianças ainda não tinham força para sugar.

*Tinha que está saindo de casa todo dia para amamentar eles e ver eles. Era uma mudança, porque todo dia tinha que estar saindo de casa para ficar com eles.* (M1)

*Está indo todos os dias ver ele, todos os dias tirar leite para ele poder mamar, todos os dias ver ele ali, isso eu tive que me adaptar muito.* (M2)

Outro desafio vivenciado foi o de passar o dia inteiro na terapia intensiva para aproveitar ao máximo a presença do filho. Nesse contexto, compreender que o filho precisava mamar várias vezes ao dia e em pequena quantidade, ficar atenta aos horários das mamadas e, ainda, acordá-lo para mamar foram mudanças que aprenderam ao longo da experiência transicional.

*Eu chegava cedo e eu passava o dia mesmo para aproveitar ao máximo que eu pudesse. Eu ia para o ponto e, às vezes, eu chegava em casa 23:00 da noite*. (M1)

*Então, tinha que ficar atenta para saber o horário certinho para ele mamar porque tinha dia que ele não chorava. Dormia que era uma beleza.* (M7)

*Hoje, eu entendo que é de pouquinho em pouquinho, mesmo que várias vezes no dia.* (M8)

Inclusive, a participante M4 mencionou a necessidade de se adaptar a estímulos para acordar sua filha, como fazer cosquinhas no pé e retirar a roupa da criança, a fim de que ela sentisse frio e, assim, acordasse para conseguir mamar.

*Então, estimulando ela, acordando, fazendo cosquinha no pé, tirando a roupa, deixando ela sentir frio, porque ela sempre dormia, pegava e dormia e eu ficava, às vezes, uma hora, até atrapalhava a rotina do hospital para ela conseguir mamar*. (M4)

A dificuldade para dormir em casa enquanto seu filho perma- necia internado na terapia intensiva também foi citado como um condicionante inibidor da experiência. O retorno para casa sem o filho foi apontado como uma situação desafiadora, já que a mãe continuava produzindo leite materno, mas não tinha o filho por perto para mamar, o que proporcionou uma transição dolorosa para ela. O sentimento de frustração aparece em distintos mo- mentos do processo de transição.

*Quando eu comecei a dormir lá, todo mundo vai embora dormir, e eu não, vou ficar aqui, aqui eu durmo melhor do que em casa, porque em casa eu não dormia.* (M20)

*A dificuldade era chegar em casa e o peito está doendo e não ter ele para mamar.* (M12)

*Chorei muito, fui embora para casa super frustrada porque que era igual ela falou. Era simplesmente ele botar a boca e ele já ia mama e não era assim.* (M13)

### Classe 5 - Dinâmica Árvore do Conhecimento e as razões maternas para amamentar

Esta classe apresentou muitas palavras ativas referentes à dinâmica utilizada no estudo, como “tronco”, “escrever”, “raiz”, “desenhar”, “colocar”, “árvore”, “caneta” e “fruto”. A recorrência desses termos no *corpus* textual ratifica a efetividade da dinâ- mica Árvore do Conhecimento para a produção dos dados. A maioria das participantes revelou que o desejo de amamentar deu-se devido à importância do AM para a saúde dos bebês, a imunidade, o crescimento e o desenvolvimento, principalmente para o sistema neurológico, assim como para a imunidade deles.

*Escrevi que eu quis muito amamentar porque é muito importante para saúde dos bebês, para o sistema neurológico. É muito importante e para saúde deles, imunidade*. (M1)

*A raiz é a questão do crescimento mesmo, porque o leite materno é importante.* (M11)

*No tronco, as mudanças que eu tive foi que eu tive que fazer muito estímulo.* (M15)

A importância dos nutrientes presentes no leite e os benefícios da amamentação para o crescimento saudável da criança também foram os motivos pelos quais as mães quiseram amamentar seus filhos. Cabe destacar que a criação de vínculo entre mãe e filho durante a amamentação surge como mais uma razão para querer amamentar. Participantes apontaram ainda o desejo de serem as melhores mães para seus filhos. No entanto, mencionaram que o fato de não amamentar exclusivamente no peito não as fazem menos mães.

*O motivo de eu querer começar a amamentar foi sempre ficar alerta sobre a importância do leite materno, que é os nutrientes que a minha bebê precisa. É bom para criação de vínculo entre mãe e filho, isso que levou querer amamentar.* (M14)

*Eu sempre quis amamentar, sempre quis. Eu acho que é uma ligação muito bonita entre mãe e filho, acho que é quando você tem certeza que é mãe.* (M17)

*Não a amamentação em si, de fato a cobrança comigo mesmo, de querer ser a mãe, porque eu não estou sendo menos mãe porque eu não estou amamentando por completo, só que isso, infelizmente, bate na cabeça da gente.* (M14)

## DISCUSSÃO

Os resultados desta pesquisa forneceram uma compreensão aprofundada do processo transicional enfrentado por mães de CRIANES durante a amamentação. A experiência de permanecer no hospital se tornou um elemento crucial para essas mães, exigindo adaptações significativas em sua rotina diária. A hospitalização infantil ocasiona mudanças drásticas na dinâmica familiar, resultando em uma experiência diferente daquela que as famílias estão acostumadas^(17)^. A necessidade de internação traz diferentes repercussões para a vida da criança e, principalmente, para seus familiares, conforme aponta a literatura, especialmente considerando o vínculo criado entre mãe e filho durante a gestação, o parto e o pós-parto^(18)^.

O empenho das mães em amamentar o seu filho com necessida- des especiais de saúde se apresentou motivado tanto pelo desejo genuíno de prover o melhor cuidado quanto pelo conhecimento dos benefícios do AM e/ou pela condição de saúde frágil da criança. Esses achados corroboram investigação em que mães, mesmo diante das dificuldades de amamentar um recém-nascido submetido à intervenção cirúrgica, demonstraram o desejo de amamentar ou fornecer apenas seu próprio leite ao filho^(19)^.

O processo de amamentar é complexo, devido às peculiaridades físicas, psicológicas e emocionais das mães. Esta transição torna-se ainda mais desafiadora diante da imaturidade fisiológica e neuro- lógica do recém-nascido, como na prematuridade. Além disso, a própria hospitalização gerou preocupações e tensões diárias para as famílias, uma vez que a maioria esperava, ansiosamente, para rever os seus filhos, além de obter informações sobre o estado de saúde e a amamentação do recém-nascido. Essa situação levou os familiares a ir com frequência ao hospital para minimizar os seus anseios, o que dialoga com a literatura(20-21), demonstrando o empenho em superar os desafios de amamentar uma CRIANES.

Durante o processo transicional, foi importante identificar e descrever as mudanças para compreendê-lo melhor^(5)^. Com base nas experiências das participantes, as mudanças observadas du- rante o processo transicional foram idas frequentes ao hospital, atraso no início da amamentação, prática de extração do leite materno, amamentação de crianças com necessidades especiais e adaptação aos horários programados de mamadas. A permanência integral no hospital, sem revezamento, também gerou cansaço físico e desgaste emocional. No entanto, as mães preferiram lidar com essa exaustão para ficar próximas de seus filhos.

Em estudo em Campo Grande, Brasil, as participantes expres- saram o desejo de estar em casa, mas passavam o dia no hospital, intensificando o estresse e o esgotamento físico e psicológico. A falta de alternativas para revezamento e a hesitação em deixar o filho aos cuidados de outros fizeram com que algumas mães permanecessem o dia inteiro na UTIN^(22)^, semelhante ao observado neste estudo. As mães também consideravam parte de seu papel materno acompanhar o filho durante a internação, acreditando que sua presença aceleraria a recuperação do recém-nascido.

Os resultados destacaram a importância do apoio dos profis- sionais do banco de leite para as mães. Outro estudo também indicou que condutas de apoio, técnicas de massagem e extração de leite, orientações e observações de mamadas, juntamente com acolhimento e auxílio no processo transicional, foram cruciais para a experiência das mães com filhos internados na UTIN^(19)^. Mesmo com a compreensão do contexto de saúde do bebê, in- cluindo equipamentos tecnológicos e profissionais especializados, destacou-se a vontade de alimentar o filho com o próprio leite, mesmo com sondas. Assim, as nutrizes mantiveram a retirada do leite materno, mesmo com os desconfortos do processo de hos- pitalização, o que também corrobora as evidências científicas^(23)^.

Embora a ordenha não seja a prática de amamentar em si, mães e profissionais consideram que a retirada do leite constituiu uma das etapas do AM, pois permite a obtenção do leite materno, seja para ser oferecido diretamente ao bebê ou processado no banco de leite para uso posterior^(24)^. Neste estudo, as mães também enfatizaram a relevância de retirar o leite materno para alimentar seus filhos com seu próprio leite enquanto não podiam amamentar diretamente.

As mães de recém-nascidos prematuros adotaram cuidados dife- rentes com seus filhos, considerando-os mais frágeis e delicados do que bebês a termo. Investigações indicam que, devido à imaturidade dos bebês, essas mães precisam seguir horários específicos de ma- madas. Como os recém-nascidos prematuros não expressam sinais claros de fome, as mães precisam respeitar os intervalos, oferecendo aleitamento a cada três horas para evitar complicações(25,26). Essas evidências corroboram os achados desta pesquisa, em que as mães se adaptaram aos intervalos curtos entre mamadas e, muitas vezes, precisaram estimular o bebê para acordá-lo.

Diante das mudanças inesperadas e dos desafios do processo transicional, algumas mães perceberam que a amamentação, que deveria ser especial e prazerosa, estava sendo muito desafiadora para o bebê. Como resultado, optaram por interromper o proces- so, apesar de estarem cientes dos inúmeros benefícios do AM. O sucesso ou fracasso da amamentação depende de vários fatores, sem um único motivo determinante. Amamentar por imposição, sem que a mãe se sinta confortável, pode ser psicologicamente prejudicial tanto para ela quanto para o filho. A amamentação não deve ser uma obrigação dolorosa, mas uma oportunidade de criar um vínculo íntimo e fortalecer os laços afetivos^(27)^.

Os resultados sugerem que o processo transicional do AM das mães de CRIANES não foi saudável para todas as participantes. Até o momento da coleta de dados, apenas 48% das crianças estavam sendo amamentadas. As mudanças inesperadas im- pactaram negativamente a transição. Portanto, profissionais de saúde, especialmente enfermeiros, devem estar atentos quando os aspectos negativos predominam os positivos, intervindo para minimizar o sofrimento emocional e promover uma transição mais leve e de qualidade para mãe e filho.

Conforme outra pesquisa, o banco de leite exerceu papel crucial no incentivo ao AM de mães que vivenciaram a UTIN, fornecendo orientações e apoio em momentos de grande vulnerabilidade para as mulheres, devido à frustração de um parto diferente do esperado, vivência do puerpério longe do filho e dificuldades na amamentação. Além disso, o banco de leite foi fundamental para a compreensão do AM e seu manejo. Os profissionais tranquilizaram as mães sobre a produção de leite, orientaram como estimular e massagear as mamas, extrair leite materno, além de compartilharem informações sobre a pega correta para evitar fissuras nos mamilo^(28)^.

Diante da complexidade da transição de amamentar as CRIANES, foi observada a autocobrança das mães em relação à amamentação. Os enfermeiros devem estar atentos às dimensões do sofrimento psíquico materno para intervir de forma empática, priorizando o bem-estar geral da díade mãe e filho. A amamentação não deve ser uma obrigação imposta, mas um ato que fortalece o vínculo emocional^(27)^. Contudo, observar o filho utilizando uma tecno- logia para se alimentar, aliado à impossibilidade de amamentar, expõe a mãe a uma realidade inesperada. O meio pelo qual o recém-nascido se alimenta torna-se um mecanismo externo, não proveniente dela. Essa experiência pode gerar sofrimento, frustração e até culpa na mãe, especialmente para aquelas que veem o AM como parte fundamental do papel materno^(23)^. Nesse contexto, surgem uma autocobrança e uma responsabilização das mães em relação ao ato de prover o sustento à criança, seja pela própria esperança criada em amamentar o bebê imaginário idealizado durante a gestação ou pelo conhecimento prévio dos benefícios da amamentação, especialmente diante do bebê real que necessita de cuidados especiais.

Dentro dos condicionantes da transição, os estigmas presentes na sociedade atuaram como inibidores da experiência^(5)^. O julgamento social de mães de crianças com necessidades especiais que não con- seguem amamentar ou que precisam complementar a alimentação foi um fator inibidor da transição, justificado pela responsabilização de ter que amamentar ao seio segundo a opinião das pessoas do contexto sociofamiliar. Diante disso, os enfermeiros devem conhe- cer o ambiente social e comunitário em que a díade mãe-filho está inserida para auxiliar na desconstrução desses julgamentos^(24)^.

A análise da experiência das mães foi favorecida pelo referencial teórico de Afaf Meleis^(29)^, que ajuda a compreender a importância do cuidado transicional dos enfermeiros, contribuindo para o amadurecimento e crescimento pessoal, buscando equilíbrio e estabilidade na experiência vivida. Assim, sugerem-se algumas intervenções de enfermagem como: acolher, oferecer cuidado humanizado e integral; compartilhar orientações sobre a im- portância do AM e dos cuidados com a criança, inclusive com adaptações diante das distintas demandas de cuidado; apoiar emocionalmente; reduzir a ansiedade e a cobrança em relação à amamentação; tranquilizar sobre a produção de leite materno; explicar a importância do uso de fórmulas infantis quando neces- sário; promover a conscientização de que a amamentação é algo positivo e saudável para a mãe e a criança; ouvir ativamente; e apoiar a mãe quando ela decidir interromper ou desistir do AM.

### Limitações do estudo

Este estudo foi conduzido em um único serviço de saúde no interior do estado do Rio de Janeiro, utilizando exclusivamente as dinâmicas da Árvore do Conhecimento do MCS com mães de CRIANES como fonte de dados. Essa abordagem específica limita a generalização mais abrangente dos achados. Portanto, destaca-se a necessidade de desenvolver novas pesquisas sobre a temática, incluindo CRIANES e suas famílias, assim como os profissionais da saúde, especialmente os enfermeiros.

### Contribuições para as áreas da enfermagem, saúde ou políticas públicas

Os achados contribuem para subsidiar o cuidado transicional do enfermeiro, a fim de facilitar o processo de transição de ama- mentar CRIANES, de modo a garantir a prática e a continuidade do AM com autonomia, confiança e qualidade na medida em que diminuam as dificuldades e os sentimentos negativos gerados pela transição. Ademais, a aplicação da Teoria das Transições de Meleis colabora com subsídios para o avanço de futuras produ- ções periódicas embasadas nesta teoria de enfermagem e para a elaboração de modelos de cuidado de enfermagem direcio- nados para famílias que vivenciam experiências transicionais de amamentar CRIANES.

## CONSIDERAÇÕES FINAIS

Os achados do estudo revelaram as adaptações e os condicio- nantes facilitadores e inibidores presentes na experiência de mães de CRIANES no processo transicional do AM. Assim, verificou-se que as participantes vivenciaram transições múltiplas, comple- xas e simultâneas, abrangendo diversos aspectos relacionados ao nascimento, prematuridade, hospitalização, necessidades especiais de saúde da criança, além das exigências constantes do ambiente hospitalar e do processo de amamentação em si, por vezes, diferenciado das outras crianças.

Muitas enfrentaram desafios emocionais, físicos e sociais ao tentar amamentar seus filhos em meio a tantas circunstâncias adversas. Reforça-se, assim, a relevância da atuação conjunta entre enfermeiros e demais profissionais de saúde junto às mães para o cuidado transicional de qualidade no AM de CRIANES, a fim de apoiar e oferecer suporte, inclusive caso a mãe decida interromper e/ou desistir deste processo (quando os condicio- nantes dificultadores sobrepõem os facilitadores).

## Data Availability

Não se aplica
